# Ferrets develop fatal influenza after inhaling small particle aerosols of highly pathogenic avian influenza virus A/Vietnam/1203/2004 (H5N1)

**DOI:** 10.1186/1743-422X-7-231

**Published:** 2010-09-15

**Authors:** John A Lednicky, Sara B Hamilton, Richard S Tuttle, William A Sosna, Deirdre E Daniels, David E Swayne

**Affiliations:** 1 Energy and Life Sciences Division, Midwest Research Institute, 425 Volker Boulevard, Kansas City, Missouri 64110, USA; 2 Southeast Poultry Research Laboratory, Agricultural Research Service, United States Department of Agriculture, Athens, Georgia 30605, USA; 3 6006 West 78th Terrace, Prairie Village, Kansas 66208, USA; 4 Present address: College of Public Health and Health Professions, University of Florida, Box 100188, 101 S. Newell Dr (HPNP bldg), Suite 2150A Gainesville Florida 32610, USA

## Abstract

**Background:**

There is limited knowledge about the potential routes for H5N1 influenza virus transmission to and between humans, and it is not clear whether humans can be infected through inhalation of aerosolized H5N1 virus particles. Ferrets are often used as a animal model for humans in influenza pathogenicity and transmissibility studies. In this manuscript, a nose-only bioaerosol inhalation exposure system that was recently developed and validated was used in an inhalation exposure study of aerosolized A/Vietnam/1203/2004 (H5N1) virus in ferrets. The clinical spectrum of influenza resulting from exposure to A/Vietnam/1203/2004 (H5N1) through intranasal verses inhalation routes was analyzed.

**Results:**

Ferrets were successfully infected through intranasal instillation or through inhalation of small particle aerosols with four different doses of *Influenza virus *A/Vietnam/1203/2004 (H5N1). The animals developed severe influenza encephalomyelitis following intranasal or inhalation exposure to 10^1^, 10^2^, 10^3^, or 10^4 ^infectious virus particles per ferret.

**Conclusions:**

Aerosolized *Influenza virus *A/Vietnam/1203/2004 (H5N1) is highly infectious and lethal in ferrets. Clinical signs appeared earlier in animals infected through inhalation of aerosolized virus compared to those infected through intranasal instillation.

## Background

Human infections caused by H5N1 highly pathogenic avian influenza viruses (H5N1) that arose from 2003-onwards have been rare as evident by only 500 cases confirmed through 5 July, 2010. However, H5N1 have a fatality rate of about 59% [1]. In ferret transmission models, the H5N1 viruses were inconsistent in transmission by direct or indirect contact exposure including respiratory droplets, but direct intranasal exposure caused morbidity and sometimes, mortality [2,3]. In contrast, the 1918 pandemic influenza virus was easily transmissible, especially human-to-human, and caused the deaths of between 20 - 40 million people worldwide for a lethality rate of 2.5%, and experimental studies demonstrated efficient transmission ferret-to-ferret by respiratory droplets [4]. The differences in transmissibility and lethality between the two viruses is not fully understood, but the use of aerosol challenge may improve our understanding of factors responsible for transmission and lethality of the H5N1 viruses.

There is limited knowledge about the potential routes and determinants required for H5N1 influenza virus transmission to and between humans, and it is not clear whether humans can be infected through inhalation of aerosolized contemporary H5N1 virus particles. Receptor distribution in the human airway is proposed to restrict efficient inter-human transmission of H5N1 influenza virus [5]. Human influenza viruses specifically recognize α2,6-linked sialic acid (SA) receptors, which are dominant on epithelial cells in the upper respiratory tract [5]. In contrast, avian influenza viruses specifically recognize α2,3-linked SA receptors, which are located in the lower respiratory tract [5,6] and are not easily reached by the large droplets (diameter of > 10 μm) produced by coughing or sneezing [7]. As reviewed by Tellier [8], various publications state that large-droplet transmission is the predominant mode by which infection by seasonal influenza A viruses is acquired by humans [7,9,10]. However, others refer to aerosols as an important mode of transmission for influenza [11-15]. It is also possible that transmission occurs through direct contact with secretions or fomites with oral, conjunctival and nasal mucus membranes because the virus can remain infectious on nonporous dry surfaces for up to 48 hours [16]. Since human infections with 2003 to present year H5N1 influenza viruses has been associated with high death rates and because healthcare workers cannot as yet be protected by vaccination, it is important to understand how the viruses can be transmitted to humans.

To date, transmission of H5N1 viruses to humans has been inefficient, occurred primarily through close contact with infected birds or, in a single case, consumption of raw infected duck blood [17]. Transmission of seasonal influenza A viruses by large droplets without accompanying aerosols has been simulated by intranasal droplet infection [18]. It is assumed that H5N1 infections may be acquired through droplet transmission routes, since intranasal inoculation of ferrets with H5N1 strains (used as a model for droplet infection) can result in clinical signs of severe influenza [3,19-22]. Whereas there is some evidence for limited human-to-human transmission of H5N1 [17,23-26], and the ferret model used as a surrogate for droplet infection suggests H5N1 infections can occur through droplets, it is still unclear whether droplet infection is the primary route of H5N1 transmission in humans. Because some of the circulating H5N1 avian viruses have demonstrated uncharacteristic affinity for α2,6-linked SA receptors and are therefore potentially dangerous to humans [27,28], it is important to evaluate their transmissibility in a suitable animal model. Domesticated ferrets (*Mustela putorius furo*) have been shown to be an appropriate animal model [29] for study of the pathogenicity [19,21] and transmissibility [30,31] of influenza viruses. On the basis of H5N1 virus cell tropism in their lower respiratory tract, ferrets have also been proposed to be a good small-animal model of human H5N1 pneumonia [6]. Since 1997, highly pathogenic H5N1 viruses have evolved into multiple genetic clades and differ in their pathogenicity to mammalian species [19,21,32-34]. For example, some H5N1 viruses spread systemically to multiple organs of inoculated ferrets [19,21,32].

We hypothesized that clinically apparent infections can arise from inhalation of aerosolized H5N1 viruses, and tested our hypothesis using inhalation exposure studies of aerosolized H5N1 in a ferret model. In this report, aerosols are defined as suspensions in air of small solid or liquid particles that remain airborne for prolonged periods of times due to their low settling velocity. Since particles ≥ 6 μm are increasingly trapped in the upper respiratory tract [35], the size cut-off of ≤ 5 μm used by many authors is also used here in reference to aerosols. Three available relatively recent H5N1s isolated from humans or animals from 2004 to 2006 that caused low to high pathogenicity in their original hosts (Table 1) were chosen for an initial assessment of pathogenicity in ferrets. Ferrets were intranasally instilled with the H5N1s. Of the three H5N1s, one was judged more virulent than the others and was aerosolized using a nose-only bioaerosol inhalation exposure system (NBIES) that we recently described and validated [36]. We report that as for intranasal instillation, inhalation of small aerosol particles of that H5N1 virus strain causes severe influenza encephalomyelitis and a lethal outcome.

**Table 1:** Virus strains used in current study and previous data on ferret pathogenicity.

			Virus pathogenicity	
			
H5N1 virus	Virus acronym	Clade **and subclade**^**a**^	Original host (reference)	Ferrets (reference)
A/Vietnam/1203/2004	VN/04	1	High^b ^[21]	High [19-21]

A/Mongolia/244/2005	MO/05	2.2	High^c ^[50]	Moderate [20]

A/Iraq/207-NAMRU3/2006	IQ/06	2.2	Mild^d ^[39]	Unknown^e^

^a^Based on the phylogenetic analysis of the HA genes [34].

^b^Fatal in 10-yr-old human male.

^c^Isolated from dead whooper swan.

^d^Mild illness in 3-yr-old human male.

^e^No known previously published data.

## Results

### 1. Pathogenicity of the H5N1 viruses in ferrets following intranasal inoculation

The pathogenicity of the three viruses differed in ferrets following droplet deposition directly into nasal cavities. Each virus was infectious at each of the intranasal (IN) doses (10^1 ^to 10^4 ^TCID_50_/ferret). A/Vietnam/1203/2004 (VN/04) caused neurological signs, temperature elevation, and weight loss (up to 21.6%) (Table 2), as previously reported [19-21]. In contrast, whereas ferrets inoculated with A/Mongolia/244/2005 (MO/05) and A/Iraq/207-NAMRU3/2006 (IQ/06) viruses developed fever, they did not develop neurological signs, and overall had lower weight losses (up to 15.6%) (Table 2). Neither MO/05 nor IQ/06 caused lethal infections and none of the animals infected by those viruses had to be euthanized for humanitarian reasons (Table 2). Viruses MO/05 and VN/04 were isolated from both nasal wash and rectal swab specimens at days 3 and 5 p.i., while IQ/06 was isolated from nasal washes but not from rectal swab specimens. The viruses isolated in Mv1 Lu cells from nasal washes and rectal swab specimens (Table 2) formed cytopathic effects typical for influenza viruses and were confirmed as influenza A viruses by first screening with commercial solid phase ELISA test (QuickVue Influenza A and B kit, Materials and Methods) followed by RT-PCR and sequencing of representative samples. Taken together, pathogenicity following intranasal inoculation was judged, as greatest to least pathogenic, as: VN/04 > MO/05 > IQ/06. From these results, VN/04 was the most virulent and chosen for aerosol studies.

**Table 2:** Outcomes of IN instillations of three different H5N1 influenza viruses in ferrets.

Virus	**Dose**^**a**^ (TCID50 units/ferret)	Clinical signs			**Inactivity index**^**e**^	Neurologic signs and related observations	**Lethality**^**h**^	**Virus titer**^**i**^		Isolation of H5N1 from rectal swab specimens on indicated day postinfection	
								
		**Maximum weight loss**^**b **^**(%)**	**Weight at termination**^**c **^**(%)**	**Maximum T increase**^**d **^**(°C)**				Nasal washes on indicated day postinfection			
								day 3	day 5	day 3	day 5
VN/04	4.9 × 101	-4.21	-3.71	1.5	2	None observed	12(t)	2	2	+	+
	
	4.9 × 10^1^	-20.03	-20.03	1.6	4	Ataxia^f^; shaking of head	12(e)	1	-	+	+
	
	4.9 × 10^1^	NA	+9.68	NA	2	None observed	12(t)	-	-	-	-
	
	4.9 × 10^2^	-2.36	-2.36	2.0	2	None observed	12(t)	2.9	3	+	+
	
	4.9 × 10^2^	-9.18	-9.18	1.4	2	Ataxia	7(e)	1.9	3	+	+
	
	4.9 × 10^3^	-0.78	+1.82	1.3	2	None observed	12(t)	1.9	1.5	+	+
	
	4.9 × 10^3^	-3.85	+5.20	0.7	2	None observed	12(t)	2.9	1.9	+	+
	
	4.9 × 10^4^	-21.64	-21.64	2.0	4	Ataxia; convulsions^g^	5(e)	4	4	+	+
	
	4.9 × 10^4^	-12.16	-12.16	3.0	2	Ataxia; convulsions	5(e)	4	3	+	+

MO/05	4.9 × 10^1^	-1.23	-0.82	1.0	1	None observed	Non-lethal	3.9	2.9	+	+
	
	4.9 × 10^1^	-4.95	No change	2.3	1	None observed	Non-lethal	6.9	5.9	ND^j^	+
	
	4.9 × 10^1^	-15.57	-10.75	2.4	2	None observed	Non-lethal	3	3	+	+
	
	4.9 × 10^2^	-3.99	+0.59	0.8	2	None observed	Non-lethal	4	5.9	+	+
	
	4.9 × 10^2^	-3.79	-2.01	1.4	1	None observed	Non-lethal	4	4	+	+
	
	4.9 × 10^3^	-5.64	-3.88	1.2	2	None observed	Non-lethal	7	8	+	+
	
	4.9 × 10^3^	-3.86	-2.45	1.1	2	None observed	Non-lethal	4	3.9	+	+
	
	4.9 × 10^4^	-13.58	-7.04	1.7	2	None observed	Non-lethal	6	4.7	+	ND
	
	4.9 × 10^4^	-12.85	-0.58	1.8	2	None observed	Non-lethal	6	5	+	+

IQ06	4.9 × 10^1^	-7.28	+3.64	0.4	1	None observed	Non-lethal	4	3	-	-
	
	4.9 × 10^1^	-0.24	+2.87	1.6	1	None observed	Non-lethal	1.9	1.9	-	-
	
	4.9 × 10^1^	-0.59	+3.4	2.0	1	None observed	Non-lethal	3	1.9	-	-
	
	4.9 × 10^2^	-1.0	+3.39	1.0	2	None observed	Non-lethal	1.9	1.9	-	-
	
	4.9 × 10^2^	-2.85	-1.83	1.9	2	None observed	Non-lethal	2	1.9	-	-
	
	4.9 × 10^3^	-0.96	+0.24	1.9	2	None observed	Non-lethal	2.9	5	-	-
	
	4.9 × 10^3^	-1.2	-1.2	2.1	1	None observed	Non-lethal	6	7	-	ND
	
	4.9 × 10^4^	-5.4	-5.44	2.1	2	None observed	Non-lethal	6	5	-	-
	
	4.9 × 10^4^	-1.16	-1.05	1.8	2	None observed	Non-lethal	6	6	-	-

^a^Based on TCID_50 _in Mv1-Lu cells.

^b,c^Compared to body weight at day 0.

^d^Compared to baseline temperature.

^e^Highest inactivity index value in one observation prior to death or euthanasia.

^f^Ataxia; incoordination and unsteadiness.

^g^Convulsions; involuntary muscular contractions.

^h^Lethality; Day ferret euthanized (e) for humanitarian reasons or terminated (t) at end of study

^i^Values for each animal are expressed as virus titers (log_10 _TCID_50_/mL) obtained using Mv1 Lu cells.

^j^ND; Not determined due to destruction of the cellular monolayer by another virus.

### 2. Virus stability in aerosol vehicle

The stability of VN/04 in aerosol vehicle (PBS + 0.5% w/v BSA fraction V) was confirmed. After one hour at room temperature, no loss of titer was detected in the presence or absence of antifoam agent B (data not shown).

### 3. Inhalation exposure of ferrets to VN/04

**Figure 1:**
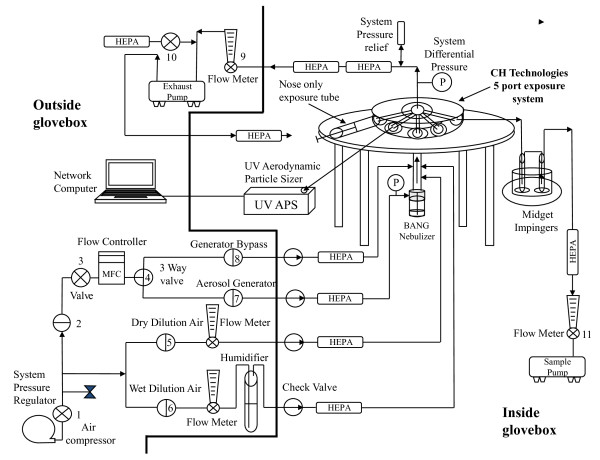
**Schematic representation of the NBIES**. Components outside (left) and inside (right) the glovebox are demarcated.

**Figure 2:**
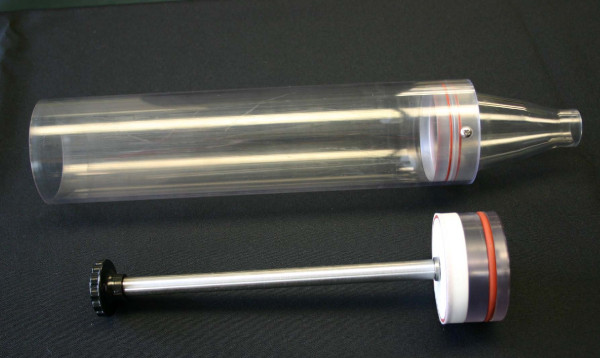
**Ferret holder**. Shown are the ferret restraint tube with integral connector cone (above) and push rod ["plunger"] (below).

An improved NBIES system, slightly modified from the original version [36] by the addition of an additional pump attached to the sampling system (Figure 1), functioned as designed without mechanical failures or perturbations of aerosol stream. Ferret holders (prototypes built for this project, Figure 2), were designed to accommodate 3-month old female ferrets. No problems were detected during inhalation exposure; the animals' faces/heads did not change color (no cyanosis or reddening of face or ears), suggesting proper oxygen intake, and other signs of stress were not observed. Previous tests verified that heat transfer from ferret body out of the restraint tubes was efficient; neither heat stress nor elevated body temperature was detected during inhalation exposure studies. Upon release from the restraint tubes, the animals resumed normal behavior without incident.

**Figure 3:**
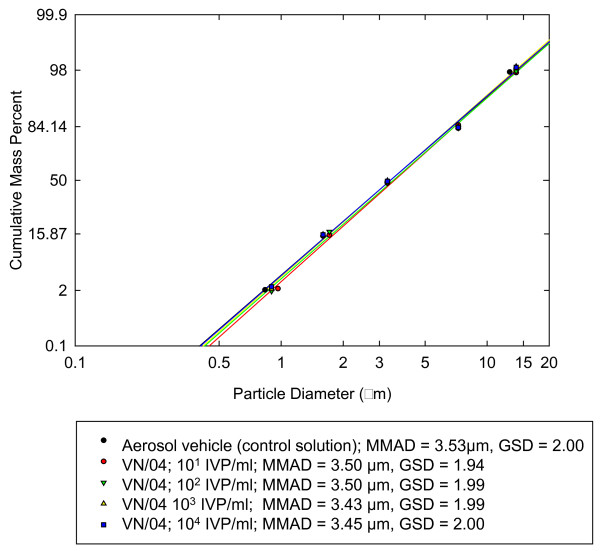
**Aerosol size log-probability plot for VN/04**. The MMAD and GSD are indicated at four different concentrations of virus and for the control solution.

Measurements of the mean mass aerodynamic diameter (MMAD) of the aerosol stream during the exposure period (10 min) were taken at 30 sec intervals using the APS. The results for all doses are summarized as an aerosol particle size log-probability plot (Figure 3). As shown, the MMAD ranged from 3.43 - 3.5 μm with geometric standard deviations (GSD) of 1.94 - 2.0 over 4 dose ranges. For aerosol vehicle (PBS + BSA) alone, the values were: MMAD of 3.53 μm, GSD = 2.

### 4. Clinical observations and pathogenicity of VN/04 in ferrets following aerosol exposure

The results of exposure to aerosolized VN/04 are summarized in Table 3. As typical for the range-finding pilot experiments performed here, the numbers of animals that were used in this work are small [19-22] but suggest that serious clinical signs occur sooner in animals exposed to aerosolized VN/04 than animals infected by the same virus through IN instillation (Table 4). Neurologic signs were also apparent in a greater % of animals. Loose stools and shedding of the lining of the large intestine were evident by day two p.i. in the aerosol group, later in the IN group. Fever and weight loss (up to -25.95%) were similar to those observed for the IN group infected with VN/04. In contrast, the negative control group that inhaled only aerosol vehicle plus antifoam agent (but no virus) remained clinically normal and achieved a normal weight gain during the course of the observation period. This indicated that neither inhalation of aerosol vehicle or antifoam B caused the morbidity and mortality in the animals exposed to aerosolized VN/04.

**Table 3:** Outcomes of exposure of ferrets to aerosolized VN/04.

**Presented dose**^**a**^ (TCID50 units/ferret)	Ferret weight and temperature			**Inactivity index**^**e**^	Neurologic signs and related observations	**Lethality**^**j**^	**Virus titer**^**k**^		Isolation of H5N1 from anal swab specimens on indicated day postinfection	
							
	**Max. wt. loss**^**b **^**(%)**	**Wt. at term.**^**c **^**(%)**	**Max. T increase**^**d **^**(°C)**				Nasal washes on indicated day postinfection			
							day 3	day 5	day 3	day 5
3.2 × 10^1^	-11.17	-11.17	1.7	2	None observed	12(e)	2	1	+	+
	
	-16.91	-16.91	0.6	4	Unresponsive (moribund)	5(e)	3	4	+	+
	
	-25.95	-25.95	1.9	4	Ataxia^f^ Convulsions^g^ Hyper-responsivness to tactile stimulus	5(e)	2	4	+	+

3.4 × 10^2^	-20.56	-20.56	1.3	2	Ataxia Convulsions Aggression-dementia^h^	4(e)	2	NA	+	NA
	
	-8.44	-8.44	1.7	3	NA	3(d)	NA	NA	NA	NA

3.4 × 10^3^	-22.59	-22.59	2.1	4	Ataxia; Hind-limb paralysis; Aggression-dementia	4(e)	3	NA	+	NA
	
	-17.31	-17.31	1.6	3	Ataxia; Convulsions	4(e)	4	NA	+	NA

3.4 × 10^4^	-17.21	-17.21	1.5	2	Ataxia; Convulsions Head tilt^i^	4(d)	4	NA	+	NA
	
	-19.40	-19.40	2.0	3	Ataxia; Head tilt	4(e)	3	NA	+	NA

^a^Based on TCID_50 _in Mv1-Lu cells.

^b^Max. wt., Maximum weight compared to body weight at day 0.

^c^Wt. at term., Weight at termination compared to body weight at day 0.

^d^Compared to baseline temperature.

^e^Highest inactivity index value in one observation prior to death or euthanasia.

^f^Ataxia; incoordination and unsteadiness.

^g^Convulsions; involuntary muscular contractions.

^h^Aggression-dementia; excessively aggressive biting and snapping of jaws at shadows, inanimate objects including cage walls, and at caretakers.

^i^Head tilt (torticollis).

^j^Day ferret found dead (d) or day euthanized (e) for humanitarian reasons.

^k^Values expressed as log_10 _TCID_50_/mL of virus titers using Mv1 Lu cells.

**Table 4:** Clinical and behavioral observations in virus-infected ferrets.

Sign/Observation		**Range of day(s) symptoms observed postinoculation with virus**^**a**^			
		
		WS/05 intranasal	IRAQ/06 intranasal	Viet/04 intranasal	Viet/04 aerosol
Death^b^		N/O^c^	N/O	Days 5 - 12	Days 3 - 5

Soft stool/diarrhea		N/O	N/O	Days 3 - 5	Days 2 - 5

Fever		Days 2 - 7	Days 2 - 7	Days 2 - 9	Days 2 - 5

Inappetence		Days 2 - 7	Days 4 - 7	Days 3 - 12	Days 2 - 5

Labored breathing^d^/wheezing		Days 4 - 6	N/O	Day 5	Days 4 - 5

Lethargy		Days 3 - 7	Days 4 - 5	Days 3 - 12	Days 1 - 5

Neurologic signs	Aggression-dementia	N/O	N/O	N/O	Day 4
	
	Ataxia	N/O	N/O	Days 5 - 12	Days 4 - 5
	
	Convulsions	N/O	N/O	Day 5	Days 4 - 5
	
	Head-tilt	N/O	N/O	N/O	Day 4
	
	Hind-limb paralysis	N/O	N/O	N/O	Day 4
	
	Shaking of head (only)	N/O	N/O	Days 11- 12	N/O

Shaking (whole body) or shivering		Days 5 - 6	N/O	N/O	Days 2 - 4

Sneezing		Days 5 - 6	N/O	N/O	Day 2

Weight loss		Days 1 - 7	Days 4 - 7	Days 2 - 12	Days 1 - 5

Resolution		Day 8 onwards	Day 8 onwards	Uncertain	N/O

Dehydration/Thin		Day 7	N/O	Days 5-12	Days 3-5

^a^Ten-day observation period for MO/05 and IRAQ/06; twelve-day for Viet/04.

^b^Animals found dead or euthanized for humanitarian reasons

^c^N/O; not observed.

^d^Labored breathing; animals exhibited open-mouth breathing with exaggerated abdominal movement.

**Figure 4:**
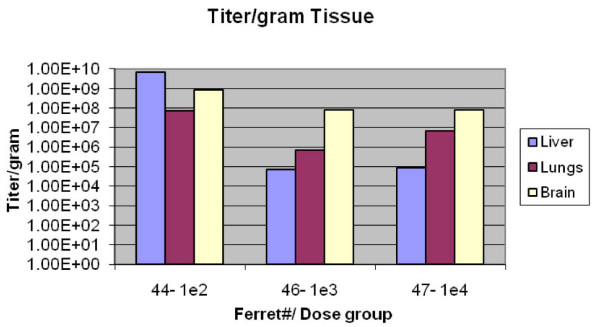
**Virus titers in brain, liver, and lung tissues taken from three animals**.

Three organs (brain, heart, and lung) chosen for virus titration were taken from three animals that received presented doses of 10^2^, 10^3^, or 10^4 ^TCID_50 _as aerosolized infectious virus particles. Higher titers were detected in brain than in lung tissues (Figure 4). Brain, heart, kidneys, liver, lungs, and spleen were also collected for histology and immunohistochemistry analyses from two animals that received 10^1 ^and 10^4 ^TCID_50 _as aerosolized infectious virus particles, from one animal instilled with 10^1 ^TCID_50 _as infectious virus particles, and one negative control animal from the aerosol and IN groups. Brain lesions and H5N1 viral antigen were found in ferrets exposed to virus by either aerosol or IN routes. The animal exposed to 10^1 ^virus particles by aerosol demonstrated evidence of systemic disease, with lesions in liver and spleen tissues at 5 days p.i. In contrast, the animal that received the same dose by IN route developed neurologic signs seven days later, but did not have liver or spleen lesions 12 days p.i. Among the three virus-infected animals for which pathology studies were performed, lung lesions were apparent only in the animal that inhaled a dose of 10^4 ^aerosolized virus particles. Interestingly, gross examination revealed external evidence of multilobar pneumonia only in the lungs of ferrets receiving doses of 10^4 ^virus particles by aerosol or IN routes, consistent with histology and immunohistochemistry results. No lesions were present in the negative control animals that were administered only PBS (IN group) or PBS + antifoam agent (aerosol group).

## Discussion

We determined that small particle aerosols of VN/04 were highly infectious in ferrets. As previously shown for IN instillation, VN/04 was neurotropic when inhaled as a small particle aerosol. At low inhaled doses (10^1 ^to 10^3 ^TCID_50 _units of VN/04/ferret), small particle aerosols of VN/04 can result in infection and resulting brain lesions without accompanying lung lesions in ferrets. In support of this notion, the titer of VN/04 in brain tissues was higher than that detected in lung tissues in animals that inhaled aerosolized virus. At a higher inhaled dose (10^4 ^TCID_50 _units of VN/04/ferret), pneumonia also occurs. This small study suggests that clinical disease appears earlier in ferrets exposed to VN/04 by aerosol versus IN routes, though severe disease resulted from both routes of inoculation.

The MMAD measurements showed consistent particle delivery (for all four dose groups) that centered on a size range that should be respired and deposited in the lower respiratory tract of humans. There was little difference in size to the MMAD of the control alone, suggesting one virus particle was trapped in the salt-BSA complex in the aerosols. There is no formal proof that the particles detected by the APS indeed contained virus (the virus may have aerosolized as free virus particles), but development of lung lesions and detection of virus in lung tissues prove delivery and deposition of virus in the lungs. In addition, the presence of brain lesions without lung lesions in the lower dose groups, suggests deposition in posterior nasal cavity and direct extension along olfactory nerves to the brain. Previously, intranasal inoculation or feeding MO/05 infected chicken meat to ferrets produced an upper respiratory infection with local extension along olfactory nerves to olfactory bulbs [20]. Similarly, intranasal inoculation with VN/04 produced abundant viral antigen in olfactory bulbs of ferrets [20].

The NBIES exposure port flow velocity (0.234 m/s) is relatively low (~0.52 m/hr or ~ 0.84 km/hr); therefore stress caused by airstream impaction on the animal's face is not an issue. Moreover, the actual volume of air in front of the animals' face (approx. 12.9 ml) is small and changes frequently relatively to the volume delivered/min for each port (*Q_port_*); thus, rebreathing of exhaled air and stalling of aerosolized viral particles should not occur. The system flow rate *Q_sys _*of 5 L/min surpasses the calculated *V_m _*for 5 animals by a factor of about 2.9× with a high estimate of 0.345 L/min for *V_m_*, and a factor of 5×; with a value of 0.2 L/min. The same values apply to air changes; at 0.345 L/min, the number of air changes required is 0.345 L/min × 5/0.101 L = ~17.1, since there are 49.4 changes/min, ~ 2.9 air changes occur per breath, showing that adequate airflow is generated. Adequate air flow is important for accurate dose calculations as well as for the reduction of stress due to the inhalation of increased CO_2 _levels that occurs when air is re-breathed.

A striking finding in this pilot study with relatively few animals is that infection acquired through inhalation exposure results in more abrupt clinical signs and may be associated with increased probability of developing neurologic disease. Since pathology examinations were performed on only three virus-infected animals, large conclusions could not be drawn over the route of exposure and pathogenesis. Some general conclusions inferred from our histology/immunohistochemistry and virology work are that: (a) histologic changes may not be present even with high virus titer in particular tissues, and (b) that brain lesions are possible without lung lesions in H5N1 infections suggesting direct extension of the virus from posterior nasal cavity through olfactory nerves into the brain, in agreement with a previous report [3]. These findings underscore the need to perform pathology analyses in conjunction with virology analyses to understand the course of H5N1 disease.

The 50% infectious dose in ferrets (FID_50_) and the FLD_50 _of VN/04 might be inferred but were not determined in this work (such a task requires many more animals). However, it is clear that the number of infectious VN/04 particles necessary to cause fatal infection is ≤ 40. From this work, it is concluded that VN/04 is highly infectious through airborne routes of infection. The extent this occurs in natural infections with viruses within the clade that includes VN/04 is unclear. Though virus was present in nasal washes, the amount of virus in the URT is low with contemporary H5N1s. Furthermore, sneezing, which primarily results in the formation of droplets, was rarely observed in the infected animals. Thus, droplet transmission may be lower than that encountered with seasonal influenza viruses. It remains unclear why ferret to ferret transmission is inefficient with this virus; perhaps the virus is not present in significant quantities in aerosols that might accompany sneezes or coughs. Current explanations for poor person-to-person transmission vary. One line of reasoning is that H5N1s do not have viral polymerase genes that function well in cells of the upper respiratory tract. For example, Hatta *et al*. [37] found that mutation of one amino acid in an H5N1 *PB2 *gene (the PB2 protein is a component of the viral polymerase complex) resulted in efficient replication of the virus in upper respiratory tract cells. Using a non-human primate model (Chinese rhesus macaque), Chen *et al*. [38] showed that pneumocytes and macrophages of the lower airway, not the ciliary epithelium of the trachea and bronchi, were the chief target cells in the lung tissue. They conclude that "predilection of the H5N1 virus to infect the lower airway suggests that the failure of the virus to attach to the ciliary epithelium of the trachea and bronchi may be a limiting factor in human-to-human transmissibility of the H5N1 virus". Taken together, tropism for cells of the LRT tract, and the rarity of sneezing/coughing in ferrets, result in poor transmissibility of the virus. This study predicts that person-to-person transmission will readily occur if H5N1 acquires the ability to replicate in the URT and is readily aerosolized or expelled in droplets.

## Methods

### Viruses

H5N1 strains A/Vietnam/1203/2004 and A/Mongolia/244/2005 were from archives of the Southeast Poultry Research Laboratory, and A/Iraq/207-NAMRU3/2006 was from the National Biodefense Analysis and Countermeasures Center (NBACC), which obtained the virus from Naval Medical Research Unit No. 3 (NAMRU-3), Cairo, Egypt [39] (Table 1). The viruses were received as low-passage stocks, and their identity verified using viral genomic sequencing. Ferrets were pre-screened and were shown to be negative for antibodies to circulating seasonal influenza viruses A/Solomon Islands/3/2006 (H1N1), A/Wisconsin/67/2005 (H3N2), and B/Malaysia/2506/2004 (all from Alexander Klimov, Centers for Disease Control and Prevention).

### *In-vitro *cell growth and manipulations

As the infectivity of the viruses in this work was higher in a *Mustela vison *(mink) lung (Mv1 Lu) cell line (validated at the Midwest Research Institute) than in the more commonly used Madin Darby canine kidney (MDCK) cell line used for influenza virus work (data to be presented elsewhere), Mv1 Lu cells were used to obtain viral titers. The Mv1 Lu cells were propagated in Modified Eagle's Medium with Earle's salts (EMEM) supplemented with L-Alanyl-L-Glutamine (GlutaMAX™, Invitrogen Corp., Carlsbad, CA), antibiotics [PSN; penicillin, streptomycin, neomycin (Invitrogen Corp.)], pyruvate (Invitrogen Corp.), non-essential amino acids (Invitrogen Corp.), and 10% (v/v) gamma-irradiated fetal bovine serum (HyClone, Pittsburgh, PA). The cells were negative by PCR for the presence of mycoplasma DNA using a Takara PCR Mycoplasma Detection kit (Takara Bio, USA, Thermo Fisher). Influenza viruses were grown in Mv1 Lu cells in serum-free EMEM otherwise supplemented as previously described plus L-1-tosylamido-2-phenylethyl chloromethyl ketone (TPCK)-treated mycoplasma- and extraneous virus-free trypsin (Worthington Biochemical Company, Lakewood, NJ) in 5% CO_2 _at 37°C (H5N1) or 35°C (seasonal viruses). The TPCK-trypsin used for this work had higher specific activity than TPCK-trypsin acquired elsewhere and therefore used at a final concentration 0.1 μg/ml. Virus preparations were harvested when cytopathic effects (CPE) typical for influenza viruses were ≥ 80% [40]. The 50% tissue culture infectious dose (TCID_50_) were calculated by the Reed-Muench method [41].

### Virus propagation in embryonating chicken eggs

Virus was propagated in the allantoic cavity of 9 to 11 day-old SPF chicken anemia virus (CAV)-free embryonating chicken eggs (ECE) (CRL) [40,42,43].

### Rapid detection of virus in tissue-culture supernatants and allantoic fluids

As needed, a commercial solid phase ELISA test (QuickVue Influenza A and B kit, Quidel Corp., San Diego, CA) was used for rapid detection of influenza A or B viruses following the manufacturer's instructions.

### Ferrets and their Pre-qualification for Studies

Studies were performed using descented, spayed 3-month-old female ferrets (0.5 - 0.9 kg) (Triple F Farms, Sayre, PA) that were housed individually in HEPA-filtered (inlet and exhaust) ventilated individual cages (Allentown, Inc., Allentown, NJ). The animals lacked signs of epizootic catarrhal enteritis, and were negative by microscopy for enteric protozoans such as *Eimeria *and *Isospora *species using fecasol, a sodium nitrate fecal flotation solution (EVSCO Pharmaceuticals, Buena, NJ). The ferrets were seronegative by a hemagglutination inhibition (HAI) assay [43] to circulating influenza B viruses and H1N1, H3N2, and the H5N1 influenza A viruses. Prior to performance of the HAI assay, the ferret sera were treated overnight with receptor destroying enzyme (RDE) (Denka Seiken USA, Inc., Campbell, CA) at 37°C to inactivate non-specific HAI activity, then heated at 56°C for 60 minutes to inactivate remaining RDE activity and complement proteins.

Room conditions for all work included 12 hr. light cycles, and an average relative humidity at 30% within a room temperature range between 64°and 84°F (17.8°to 28.9°C). The animals were fed pelleted ferret food (Triple F Farms) and watered *ad libitum*, and housed and maintained under applicable laws and guidelines such as the Guide for the Care and Use of Laboratory Animals (Institute of Laboratory Animal Resources, Commission on Life Sciences, National Research Council, National Academic Press, 1996) and the U.S. Department of Agriculture through the Animal Welfare Act (Public Law 89-544 and Subsequent Amendments), and with appropriate approvals from the Midwest Research Institute Animal Care and Use Committee. Body temperatures were measured twice daily via subcutaneously implantable programmable temperature transponders (model IPTT-300, Bio Medic Data Systems, Seaford, DE) implanted in the neck.

### Intranasal inoculation studies

Procedures based on those of Zitzow *et al*. [22] were used. Briefly, twelve ferrets were used for each virus study: nine (n = 9) for virus infection, three (n = 3) for non-infected controls. Ferrets were anesthesized by intramuscular administration of ketamine HCl (25 mg/kg)-xylazine (2 mg/kg)-atropine (0.05 mg/kg), and instilled with selected doses of viruses in isotonic phosphate buffered saline (PBS) with 0.5 % purified bovine serum albumin (to stabilize the viruses) and antibiotics. Fifty μl of virus suspension was instilled into each nostril (100 μl of virus suspension/ferret). Two ferrets each were inoculated IN with 10^4^, 10^3 ^and 10^2 ^TCID_50_, and three ferrets each with 10^1 ^TCID_50 _of virus (TCID_50 _values determined in Mv1 Lu)_. _A back-titration was performed on the virus doses to verify viral titers per dose. Three animals served as controls and received IN doses of a 1:30 dilution of sterile, non-inoculated chicken allantoic fluid in PBS. All the animals were caged individually and weighed once daily for the duration of the study. Body temperatures were recorded twice daily from conscious animals that were stimulated and active for at least five minutes (as there is a relatively large variance in the resting and active temperatures of ferrets). A temperature increase ≥1.4°C over baseline was considered significant; the baseline was the average temperature for the entire group over the pre-dose observation period.

Nasal washes and rectal swab specimens were collected at 3 and 5 days post-inoculation with virus. Clinical signs including sneezing (before anesthesia), inappetence, dyspnea, and level of activity were assessed daily for the duration of the study (8 - 10 days). Inappetence was judged through visual observation of the food remaining in the feeder and spilled within the surrounding area. A scoring system (relative inactivity index [RII]) based on that described by Reuman *et al*. [44] and as used by Govorkova *et al. *[19] and Zitzow *et al. *[22] was used to assess the activity level as follows: 0, alert and playful; 1, alert but playful only when stimulated; *2*, alert but not playful when stimulated; and 3, neither alert nor playful when stimulated. They were also monitored daily for nasal and ocular discharge, neurological dysfunction, and semi-solid or liquid stools. Neurologic dysfunction was defined as development of motor dysfunction (including paralysis or posterior paresis), convulsions, ataxia, seizures, and depression. Ferrets with > 25% loss of body weight or with neurologic dysfunction were anesthetized by intramuscular administration of ketamine HCl (25 mg/kg)-xylazine (2 mg/kg)-atropine (0.05 mg/kg), then euthanized with Beuthanasia-D Special (sodium pentobarbital and phenytoin sodium) or equivalent (Euthasol) via the jugular vein.

### Collection of nasal washes and virus titration

Nasal washes were collected at the same time as rectal swab specimens (one collection of each/day) after anaesthesia with ketamine (25 mg/kg) essentially as described by Zitzow *et al*. [22]. Briefly, 500 μl sterile isotonic PBS containing 1% bovine serum albumin, and penicillin (100 U/ml), streptomycin (100 μg/ml) and gentamicin (50 μg/ml) was administered (250 μl/nostril) to induce sneezes in ketamine-anaesthesized ferrets on days 3 and 5 post-inoculation with virus. Sneezes were collected in a Petri dish, and diluted to 1 ml with cold PBS containing antibiotics. A 100 μl aliquot of the diluted material was inoculated into a T25 flask containing Mv1 Lu cells and incubated to screen for the presence of H5N1 virus, and the remainder stored at -80°C. Samples positive for H5N1 viruses by the screen were then titrated for five days in Mv1 Lu cells in 96-well microtiter plates.

### Collection of rectal swab specimens and virus detection

Rectal swab specimens were collected at the same time as nasal washes (one collection of each/day) after anaesthesia with ketamine (25 mg/kg) [22]. Flocked nylon swabs paired with Universal Transport Medium (UTM) (both from Copan Diagnostics, Inc., Murrieta, CA) were used to collect and transport anal swab specimens. The swabs were pre-moistened with sterile PBS prior to specimen collection from sedated animals, inserted approximately 0.5 inches (~1.3 cm) into the rectum, retracted, then swirled in 1 ml of UTM in the transport tube. The transport tubes were vortexed for 1 minute to emulsify the fecal material in UTM. The emulsified material was diluted 1:10 in serum-free complete EMEM with trypsin, 5× PSN and Fungizone (amphotericin B) (Invitrogen), and 0.5% w/v purified BSA fraction V, and left at room temperature for 1 hr to allow the fecal solids to settle and the antibiotics to suppress bacteria and fungi. The liquid above the settled solids (nearly 10 ml) was then added to Mv1 Lu cells in T75 flasks and incubated for 1 hr at 37°C. Thereafter, 15 ml of serum-free media containing trypsin was added. Due to specimen variability inherent with the procedure, no attempts were made to quantitate the virus in the rectal swab specimens; only virus isolation was attempted. However, previous work (performed by us) indicated the titer of VN/04 was > > MO/05 in rectal swab specimens.

### Collection and virus titration of organs

Selected organs were collected for virus titration from animals that were humanely euthanized after exhibiting neurologic signs or at the time of the animal's death. Pooled organ tissue samples were collected from each of the six lobes present in ferret lungs, from all parts of the brain, and from the liver. All tissues were snap-frozen on dry ice upon collection and stored at -80°C until they were assayed for virus content/quantity. Tissue samples were weighed and ~0.5 g homogenized in sterile PBS with antibiotics and 0.5% w/v purified BSA fraction V to form a 10% w/v homogenized suspension. The homogenates were titrated for five days in Mv1 Lu cells in serum-free media with trypsin to determine the log_10 _TCID_50 _per gram of tissue. The lower detection limit was estimated at 10^1.3 ^TCID_50_/gr tissue.

### Exposure system and generation of aerosols

A nose-only bioaerosol inhalation exposure system (NBIES) assembled in a Class III IsoGARD^® ^Glovebox (The Baker Company, Sanford, ME) in an ABSL3+ laboratory was used for this work [36] (Figure 1). A nose-only system was chosen for this study over whole-body and other exposure routes because: (a) it minimizes infection by non-inhalation routes, (b) reduces requirements for post-exposure decontamination of animals (such as by wiping exterior of conscious animal with bleach), (c) minimizes potential contamination of animal housing areas, (d) lessens contamination risks for animal care personnel, and (e) permits testing at high virus concentrations while minimizing quantities of starting material. The latter consideration is important for cradle to grave work with select agents, wherein experiments are preferentially designed to utilize small amounts of agent.

Ferret restraint tubes with push rods (prototypes built for this work by CH Technologies, USA, Westwood, NJ) (Figure 2) were used along with a model 3314 Aerodynamic Particle Sizer^® ^(APS) Spectrometer (TSI Inc. St. Paul, MN). The APS is used to measure the aerosol size distribution in the test atmosphere and is operated with Aerosol Instrument Manager software, release version 8.0.0.0 (TSI, Inc.) run in a Dell Latitude D600 computer. A 3-jet BioAerosol Nebulizing Generator (BANG), (BGI Inc., Waltham, MA) was used (CH Technologies). The BANG is a low flow, low dead space nebulizer designed to operate in the range of 1 to 4 liters per minute with a pumped fluid (recirculated) flow that features minimal sample utilization. It was chosen over other nebulizers as the most appropriate generation device for the aerosolization of influenza virus; considerations included: minimal potential damage to agent, reduced clumping of virus, uniformity of droplet size distribution, and efficiency (the amount of virus that needs to be prepared is much smaller than that required by similar aerosol generators).

The exposure system contains sampling ports that are tapped for: (a) measurement of aerosol particle size, and (b), sample collection to assess live-agent aerosol concentration. Up to three animals were exposed per experiment [36]. An exposure time of 10 minutes was used [36]. Total flow through the inhalation system was 5 liters per min during the exposures created by the BANG. The metrics for using the BANG generation devices in association with the inhalation system was previously described [36]. Exposure concentration expressed in TCID_50_/ml was determined by sampling of the aerosol stream using two model 7531 midget impingers (AGI; Ace Glass Incorporated, Vineland, NJ) connected in series.

The dynamic air flow through the aerosol delivery ports on the system exceeds 3× the total ventilation volume of all animals exposed. Influenza virus is mixed with a non-toxic vehicle (sterile PBS solution with 0.5% purified BSA fraction V) to help maintain viability of the virus and act as a vehicle to generate the test aerosol. The saline solution is well characterized and its acute inhalation toxicity known; it does not cause an acute inflammatory response or stimulate excess mucus secretion leading to increased mucociliary clearance. Thus, the ferrets remain susceptible to challenge infection when the saline solution is inhaled in the quantities used in this work (J. Lednicky, unpublished). The exposure system is operated dynamically at negative pressure.

Prior to live-agent work, the aerosol system was characterized to assess individual parameters, including exposure port to port aerosol homogeneity, aerosol concentration ramp up, concentration stability and decline, sample measurement, exposure location to exposure location variation, and sampling system collection efficiencies [36].

### Calculations for aerosol transmission studies

The presented dose *D *(defined as the inhaled dose estimated from the multiplication of the aerosol concentration and the total volume of air breathed in by the animal) is estimated from the ferret respiratory rate and duration of aerosol exposure. By convention used in aerobiology, $(t_{exp})=\int_{0}^{exp}R(t)C(t)f(t)dt$, where *R *refers to respiration rate, C refers to the concentration of aerosolized agent, *f(t) *= % of agent deposited in the lungs, and *t_exp _= *exposure duration time. When the following assumptions are made: a constant minute volume (V_m_) for *R(t)*, a constant live-agent aerosol concentration (integrated air sample determined concentration for *C(t)*, 100% deposition for *f(t)*, and *t(exp) *is fixed at the time of exposure, then: *D = R × C × t_exp_*.

The ferret respiratory minute volume (V_m_), defined as the volume of air inhaled or exhaled over a minute, was estimated using Guyton's formula [45], where BW = body weight in gr, and the volume calculated in ml:

$${\text{V}}_{\text{m}}=2.10\times {\text{BW}}^{3/4}=2.10\times {\text{BW}}^{0.75}$$

Ferrets in this work ranged from about 500 to 900 gr. For a 500 gr ferret, log_10_BW^3/4 ^= 0.75 × log_10_500 = 2.02. The antilog of 2.02 = 105.7; therefore, V_m _= 2.10 × 105.7 = 222.0 ml/min (0.22 L/min). Similarly, for a 900 gr ferret, V_m _= 345.1 ml/min.

Since most of the ferrets were close to 500 gr, an *approximate *V_m _value of 0.2 L/min was used for this work. The V_m _value of 0.2 L/min used in this work was consistent with estimates obtained by multiplying the ferret tidal volume (V_t_) expressed in ml × the breathing rate (BR) of conscious ferrets expressed as breaths/minute (bpm). By definition, V_t _= the volume of air inspired or expired with each normal breath, whereas BR = number of breaths/minute (bpm) for a conscious ferret. For ferrets, V_t _= 6.06 ± 0.30 ml, and BR = 33 - 36 bpm [46,47].

$${\text{V}}_{\text{m}}={\text{V}}_{\text{t}}\times \text{BR}$$

Using an average V_t _value of 6.06 ml and an average BR of 34.5 bpm, V_m _= 209.01 ml/min = 0.21 L/min.

Precision in calculations of aerosol concentrations and estimates of the number of viruses inhaled per experiment depend largely on the collection efficiency/efficacy of the impinger(s). Therefore, the impinger system must first be characterized to establish operational parameters determined to obtain the required *D*. Systems similar to ours are often designed with a single impinger and are operated with the assumption that > 90% of the aerosolized microorganisms are entrained during sampling of the aerosol flow through the impinger. If the true efficiency is < 90%, a significant undercount of the aerosol concentration can result, and this causes both an underestimate of the inhaled dose and an overestimate of virulence (since the number of organisms to cause an infection is undercounted). Moreover, the collection fluid in the impinger must maintain the aerosolized agent in a viable (infectious) manner and quantification should be for viable agent. Otherwise, quantification of aerosolized agent based solely on biochemical or immunological assays (such as PCR or ELISA) may confound understanding by measuring both live and inactivated agents. The NBIES was designed with a dual impinger arrangement based on our previous experience: aerosolized VN/04 is not collected with high efficiency with one impinger alone under the conditions we used, whereas some seasonal influenza viruses can be (data not shown). The collection fluid (PBS + 0.5% w/v purified BSA fraction V) was validated for this work (data not shown). The lengthy steps and procedures to determine impinger collection efficiency will be presented elsewhere. After establishing conditions resulting in > 90% collection of live agent at the impingers, calculations based on (theoretical) 100% efficacy of aerosol dissemination are derived to set operating parameters:

(1) Assuming 100% efficiency, the quantity of aerosolized virus particles (VP) for a given *C_s _*is calculated as:

$$\text{VP}=C_{s}\times Q_{mist}\times t_{exp}$$

(2) The conc. of virus in impinger A is determined for a given *C_s_*

(3) The conc. of virus in impinger B is determined for the same *C_s _*in step 2

(4) The volume sampled by both impingers (*V_i_*) is calculated for *t_exp _*(for this work, 1 L/min × 10 min = 10 L)

(5) Assuming even dissemination by the system, the apparent concentration of virus (*C_app_) *in the aerosol stream is calculated as:

$$C_{app}=\text{sum of virus recovered in impingers }(\text{A}+\text{B})/V_{i}$$

(6) The volume disseminated by the system (*V_s_*) is calculated as:

$$V_{s}=\text{System flow rate}\times t_{exp}$$

(7) At 100% efficiency, the concentration of VP in the aerosol stream (*C_aero_*) is: VP/*V_s_*

(8) The true efficiency (expressed as %) of the system is: *C_app_*/*C_aero _*× 100

(9) *D *= *C_app _*× *V_m _*× *t_exp_*

Once the above are established, calculations typically used in aerobiology can be made. The concentration of virus in the aerosol stream, calculated from the virus collected in impingers 1 and 2, where *Q*_*agi*1+2 _is the collection flow rate in L/min through impingers (*agi*) 1 and 2, is:

$$C_{aero}=\frac{\left[{\left(C\times V\right)}_{agi1}+{\left(C\times V\right)}_{agi2}\right]}{Q_{agi1+2}\times t_{exp}}$$

The spray factor (*SF*), defined as the ratio of aerosol concentration to starting concentration, is a unitless measure used to predict aerosol concentration for a starting solution. A *SF *is calculated for a range of starting concentrations using the same nebulizer and flows designed for the aerosol challenge; as:

$$SF=\frac{C_{aero}}{C_{neb}}$$

where *C_neb _*= concentration of starting solution in the BANG reservoir. An average spray factor *SF_avg _*is then determined from a range of virus concentrations. The predicted respiratory volume during exposure (*V_e_*) is calculated as:

$$V_{e}=V_{m}\times \text{duration of exposure }(t_{exp})$$

The aerosol concentration (*C_aero_*) needed to attain *D *is calculated as: *V_e_C_aero _= D*

The starting concentration *C_s _*is then calculated from the value calculated for *SF_avg _*as:

$$SF_{avg}=\frac{C_{aero}}{C_{s}}$$

The system displacement volume, *V_tot_*, which is the volume of aerosolized material leaving the nebulizer/unit time, was approximated using the formula below, where *d *refers to inner diameter of the tube/cylinder and *l *is the length:

$$\text{V}tot=\sum_{i}\Pi \frac{d_{i}^{2}}{4}l_{i}$$

The velocity of air at the animals' nose (the exposure port aerosol flow velocity) was calculated as:

$$Exposure\;port\;aerosol\;flow\;velocity=\frac{Q_{port}}{A}$$

Finally, the number of system (total) air changes was calculated as:

$$Air\;changes=\frac{Q_{sys}}{V_{tot}}$$

### Aerosol exposure studies

Virus was diluted to the appropriate concentration in aerosol vehicle (PBS + 0.5% BSA fraction V), to which was added antifoam 0.25% (v/v) molecular-grade antifoam agent B (Sigma-Aldrich, Inc., St. Louis, MO). After mixing, 4 ml of virus + antifoam was placed in the reservoir. Similarly, 10 ml of PBS + 0.5% BSA fraction V but with 0.5.% (v/v) antifoam agent B was placed into each impinger. Conscious ferrets were used for inhalation studies. As for the intranasal inoculation studies, ferrets were exposed to aerosolized viruses to attain delivered doses of 10^1^, 10^2^, 10^3^, or 10^4 ^infectious virus particles over a 10 minute exposure period. All work was performed expeditiously to minimize stress; animals were moved in and out of the ferret restraint tubes relatively quickly. Just prior to exposure, the animals were loaded into ferret restraint tubes and quickly transported to the Class III glovebox housing the NBIES. The tubes were affixed onto designated inhalation ports, the aerosol generated, and the animals exposed according to experimental design.

Upon completion, the tubes were disengaged and placed in a transport bucket. The bucket was sealed, its outsides decontaminated, removed from the glovebox, and transported within the ABSL-3 suite to an animal room, where the tubes were removed within a BSC. The ferrets were then removed from the tubes and placed in cages (1 animal/cage) within the BSC, and the cages thereafter stacked in racks. Following aerosol exposure, cage-side observations including evaluation of mortality, moribundity, general health and morbidity were performed once daily during the pre-clinical stage and twice daily (at approximately 8-hour intervals) after symptoms of influenza had developed. Weight and temperature were determined once daily.

### Necropsy

All procedures were performed in an ABSL3+ laboratory. For scheduled necropsies, animals were anesthetized then humanely euthanized as described above by trained technicians. After confirming death, the animals were prosected within a Class II A2 BSC. Following gross evaluation, tissues and organs were collected in this order spleen, kidneys, intestines, liver, heart, lungs, brain. To reduce hazards, a rotary saw was not used to excise the whole brains from skulls. Instead, Dean bone side-cutting forceps were used (Robosz Surgical Instruments Company, Gaithersburg, MD), and the skull cut from back to front along the medial suture lines using long (> 5 mm) cutting strokes. Noteworthy, fragmentation and production of airborne bone chips were common when other cutting tools were used, especially with short (< 2 mm) cutting strokes.

### Histology analysis

Organs and tissues (brain, lungs, liver, spleen, heart, kidney) were collected, sliced to ≤ 5 mm in thickness, fixed in 10% neutral buffered formalin for 10 days to preserve the tissues and inactivate the H5N1 viruses, then embedded in paraffin for subsequent histology and immunochemistry examinations. Formalin-fixed and paraffin-embedded tissue sections were stained with hematoxylin and eosin for histological evaluation, and adjacent sections analyzed by immunohistochemistry with a primary antibody that recognized the influenza A nucleoprotein of H5N1 viruses as previously described [48].

### Bio-containment facilities

*In-vitro *and *in vivo *experiments with H5N1 viruses were conducted in USDA-approved biosafety level 3-enhanced (BSL3+) and animal biosafety level 3-enhanced (ABSL3 +) containment facilities, respectively, and required use of personal protective equipment and occupational health monitoring program.

### Reverse transcription-PCR and virus sequencing

Viral RNAs were isolated from allantoic fluid or cell culture supernatant (QIAamp Viral RNA kit; QIAGEN) and two-step reverse transcription-PCR was done with synthetic universal and other oligonucleotide primers [43,49]. The sequences were analyzed using an Applied Biosystem 3130 DNA analyzer by using BigDye Terminator (v. 3.1) chemistry and the same oligonucleotide primers used for amplifications.

## Competing interests

The authors declare that they have no competing interests.

## Authors' contributions

JAL conceived the overall NBIES design including choice of BANG nebulizer (with the assistance of all parties mentioned in the Acknowledgments section), interpreted data, established calculations, provided oversight, and co-prepared the manuscript; SBH established accurate virus quantification procedures, performed in-vitro virus work, assisted with data interpretation, and helped write the manuscript; RST helped design and assemble the NBIES, established protocols for inhalation exposure studies, helped with calculations, led efforts on the testing and validation of the NBIES, assisted with data interpretation, and co-wrote the manuscript; WAS assisted with the assembly, testing, and validation of the system, and oversaw animal work; DED assisted with program management, in-vitro virus work, and data interpretation; DES provided virus strains VN/04 and IQ/06, technical assistance, helped in the design of experiments, performed histopathology and immunohistochemistry evaluations, assisted in interpretation of the data, and co-wrote the manuscript. All authors read and approved the final manuscript.
